# Nurses Resilience in Mental Health Settings: Foundation for Professional and Humanistic Care

**DOI:** 10.1192/j.eurpsy.2026.10957

**Published:** 2026-07-31

**Authors:** R. Kigli, S. Rosenthal, S. Beilin

**Affiliations:** 1 Nursing, Tel Aviv University, Tel Aviv; 2 Management; 3 Nursing, Merhavim Mental Health Center, Be’er Yakov, Israel

## Abstract

**Introduction:**

Recent years in Israel have presented unprecedented emotional, professional, and national challenges, necessitating renewed examination of healthcare professionals’ resilience capabilities. Personal resilience among nursing staff significantly influences therapeutic environment construction, clinician experience, and ultimately, patient outcomes in mental health settings.

**Objectives:**

To develop and implement a comprehensive resilience enhancement program for mental health nurses, strengthening individual and team resilience capacities through interventions and practical tools for effective coping with uncertainty, emotional burden, and crisis situations.

**Methods:**

This multi-phase intervention study targeted nursing management teams initially, followed by ward-level nursing staff at a Israel’s largest mental health center. The program incorporated two validated theoretical models: Robertson and Cooper’s “Four Sources of Personal Resilience” (Robertson et al. Leadership Organ Dev J 2012; 33:224-232) and Prof. Molli Lahad’s BASIC PH model (Leykin et al. Jessica Kingsley 2012). Implementation phases included: (1) steering committee formation and program design; (2) customized intervention development based on literature review and clinical expertise; (3) pilot session with nursing leadership; (4) structured workshops for charge nurses; (5) department-wide implementation beginning with sexual trauma units; (6) continuous evaluation and learning. Resilience assessment utilized a validated personal resilience questionnaire (Connor & Davidson. Depress Anxiety 2003; 18:76-82).

**Results:**

The pilot leadership session demonstrated enhanced individual and team resilience among management staff, providing crucial feedback for program refinement. Two comprehensive workshops for charge nurses successfully introduced theoretical concepts through experiential activities and practical exercises. Participants showed increased resilience awareness and acquired concrete tools for daily implementation. Initial implementation in specialized trauma units revealed high engagement levels and positive reception. Ongoing evaluation demonstrates improved coping strategies and enhanced sense of professional efficacy among participating nurses.

**Conclusions:**

This innovative resilience program created meaningful, empowering experiences for both facilitators and participants, generating curiosity, shared learning, and professional growth. The intervention extends beyond workshop sessions, providing sustainable tools for ongoing practice through annual programming to maintain resilience as a core organizational value. Results suggest that structured resilience interventions can significantly enhance nursing staff capacity to manage complex clinical situations while maintaining quality patient care. The program offers a replicable model for mental health organizations seeking to support staff wellbeing and therapeutic effectiveness.

**Disclosure of Interest:**

None Declared

